# Clear Cell Adenocarcinoma of the Urethra: Review of the Literature

**DOI:** 10.1155/2015/790235

**Published:** 2015-01-20

**Authors:** Anthony Kodzo-Grey Venyo

**Affiliations:** North Manchester General Hospital, Department of Urology, Manchester, UK

## Abstract

*Background.* Clear cell adenocarcinoma of the urethra (CCAU) is extremely rare and a number of clinicians may be unfamiliar with its diagnosis and biological behaviour. *Aims.* To review the literature on CCAU. *Methods.* Various internet databases were used. *Results/Literature Review.* (i) CCAU occurs in adults and in women in the great majority of cases. (ii) It has a particular association with urethral diverticulum, which has been present in 56% of the patients; is indistinguishable from clear cell adenocarcinoma of the female genital tract but is not associated with endometriosis; and probably does not arise by malignant transformation of nephrogenic adenoma. (iii) It is usually, readily distinguished from nephrogenic adenoma because of greater cytological a-typicality and mitotic activity and does not stain for prostate-specific antigen or prostatic acid phosphatase. (iv) It has been treated by anterior exenteration in women and cystoprostatectomy in men and at times by radiotherapy; chemotherapy has rarely been given. (v) CCAU is aggressive with low 5-year survival rates. (vi) There is no consensus opinion of treatment options that would improve the prognosis. *Conclusions.* Few cases of CCAU have been reported. Urologists, gynaecologists, pathologists, and oncologists should report cases of CCAU they encounter and enter them into a multicentric trial to determine the best treatment options that would improve the prognosis.

## 1. Introduction

Clear cell adenocarcinoma of the urethra (CCAU) is rare in both sexes but has been more commonly described in the female urethra. Even in the female CCAU is very rare. Information regarding CCAU has been obtained from single case reports and small case series [1, 2]. The ensuing paper contains a review of the literature which has been divided into (A) Overview which has broadly summarized CCAU and (B) Discussion and narrations from reported cases and case series of CCAU.

## 2. Methods

Various internet search databases were used to obtain literature on CCAU using the following key words: clear cell adenocarcinoma of urethra; renal cell carcinoma of urethra; primary; metastatic; secondary. Twenty-six references were identified which were suitable for the review of the literature.

## 3. Literature Review

### 3.1. Overview

#### 3.1.1. General

*Epidemiology.* Clear cell adenocarcinoma of the urethra most commonly occurs in women with a mean age of 58 years (range 35 to 80 years) [3].

*Aetiology.* CCACU is conjectured to arise from surface urothelial metaplasia or Müllerian rests or Müllerianosis [4].

#### 3.1.2. Presentation

CCACU tends to have similar clinical manifestation to the other urethral carcinomas [1, 3], haematuria [2].

#### 3.1.3. Investigations

*Urine Cytology.* Patients tend to present with haematuria and when they are first seen their urine specimens are sent for cytological examination in addition to the urine specimens being sent for microscopy and culture.

*Cytological Features.* The cytological features of CCAU include: (i) enlarged tumour cells which contain abundant clear cytoplasm with conspicuous vacuoles; (ii) hobnail patterned cells; (iii) and hyaline globules.

*Urethrocystoscopy*

- Urethrocystoscopy enables the surgeon to visualise the urethral tumour and provides a means by which biopsies are taken for histological examination to establish the diagnosis of CCACU.
- Examination under anaesthesia at urethrocystoscopy enables the surgeon to bimanually examine and assess the urethral tumour for fixity of the tumour and to determine how easy or difficult it might be to completely excise the lesion at operation.

#### 3.1.4. Radiological Imaging

The following radiological investigations can be used to localize a mass in the urethra as well as show whether there is any urinary bladder wall thickness, pelvic lymph node involvement, or distant metastases.

Ultrasound scan may reveal urethral mass [5].

MRI scan may reveal urethral diverticulum containing a nodular enhancing malignancy [6] or a heterogeneous mass in the urethra [5].

CT scan may reveal urethral diverticulum containing a heterogeneous mass [5, 6]. It could be imagined that if there is no urethral diverticulum the CT scan may demonstrate urethral mass only.

Isotope bone scan can also reveal whether or not there is bony metastasis [5, 6].

#### 3.1.5. Macroscopic Features

Most commonly (56%) CCACUs are found as tumours arising in urethral diverticulum [3].

For microscopic features, see Figures 1, 2, 3, 4, 5, 6, 7, 8, 9, 10, 11, 12, and 13 which show various microscopic and immunohistochemical staining characteristics of the tumour.

- The microscopic characteristics of CCAU are similar to clear cell adenocarcinoma of female genital tract.
- CCAUs tend to exhibit the classic triad of (a) tubulocystic, (b) papillary, and (c) diffuse patterns [2, 3] which characterize the tumour.
- Microscopic examination of CCAUs shows hobnail and flattened cells with abundant clear cytoplasm, moderate to marked nuclear pleomorphism, and frequent mitotic figures are seen [2, 3].

For immunohistochemical staining characteristics, see Figures 1 to 13 which show various microscopic and immunohistochemical staining characteristics of the tumour.

#### 3.1.6. Positive Staining

CCAUs exhibit positive immunohistochemical staining for

- PAX2 [3],
- PAX8 [3],
- cytokeratin 7 [5, 9],
- p16 [5],
- p53 [5],
- CA125 [5],
- CAM5.2 [5],
- AE1/AE3 [5].

#### 3.1.7. Negative Staining

CCAUs exhibit negative immunohistochemical staining for

- PSA [2, 3],
- PAP [3],
- thrombomodulin [5],
- oestrogen [5],
- progesterone [5],
- cytokeratin 20 [5],
- p63 [5],
- CD10 [5],
- CEAP [5],
- WTI [5],
- AFP [5],
- s100 [5].

#### 3.1.8. Differential Diagnoses

- Metastatic involvement from the female genital tract should be excluded [4].
- Nephrogenic adenoma in which no marked nuclear pleomorphism can be seen on microscopy, in which no mitotic figures can be seen, and in which there exists no infiltrative or solid growth pattern.

#### 3.1.9. Treatment

- A small urethral tumour of CCAU may be effectively treated by urethrectomy alone.
- Urethrectomy in conjunction with cystoprostatectomy or urethrectomy in combination with anterior exenteration would be considered good options of treatment for CCAU.
- Anterior exenteration and pelvic lymph node dissection were the treatment used in most reported cases of CCAU (as in [5, 6, 9]).
- Consolidation radiotherapy to the pelvis had been given in a case of pelvic lymph node involvement.
- There is lack of knowledge of the effectiveness of chemotherapy in the treatment of CCAU and there has not been any documentation to suggest that chemotherapy is effective in the treatment of CCAU (see [10] in which chemotherapy was given).

#### 3.1.10. Prognosis

Few cases of CCAU have been reported and it would appear so far that CCAU is an aggressive tumour with low 5-year survival rates. There is therefore the need to explore for treatment options that would improve the prognosis.

### 3.2. Discussion and Miscellaneous Narrations from Some Reported Cases and Case Series

Oliva and Young [3] reported 19 clear cell adenocarcinomas of the urethra in 1996. They reported that out of the 19 patients with CCAU 18 were from women and 1 from a man. The ages of the patients ranged from 35 years to 80 years and the average age was 58 years. They stated that the clinical manifestation and macroscopic findings were similar to those of urethral carcinomas, except for the fact that 12 tumours which were all found in women arose within urethral diverticulum. They also reported that microscopic examination revealed that the neoplasms exhibited the classic triad of (a) tubulocystic, (b) papillary, and (c) diffuse patterns which characterized the tumour. Furthermore, they reported the following.

- The tumours exhibited the typical cytological characteristics of clear cell adenocarcinoma which included hobnail cells, flattened cells, and cells with abundant clear cytoplasm.
- Nuclear pleomorphism was typically at least moderate and was marked in almost half of the specimens.
- They easily found mitotic figures in almost all the specimens.
- The aforementioned cytological characteristics should be helpful in the distinction of CCAU from benign nephrogenic adenoma, even though one of their patients was initially misdiagnosed as having nephrogenic adenoma.
- They had performed immunohistochemical staining of the tumours for prostate-specific antigen (PSA) and prostatic acid phosphatase (PAP) on 13 tumours and all were negative.
- Follow-up was available for 13 patients. Six of the patients did not have any evidence of recurrence up to 10 years postoperatively. Four patients had died of disease from between 5 months and 42 months postoperatively. Three more patients developed recurrence but they were alive up to 6.5 years following their presentation.

Scantling et al. [6] reported a 47-year-old woman with a history of chronic recurrent urinary tract infection who was diagnosed with urethral carcinoma during investigation for visible haematuria, hesitancy, straining, and urge incontinence. She had cystoscopy which revealed a papillary urethral mass emanating from urethral diverticulum, and histological examination of the biopsy specimen revealed clear cell adenocarcinoma. She had a contrast enhanced magnetic resonance imaging (MRI) scan and computed tomography (CT) scan of the pelvis which confirmed the urethral diverticulum containing a nodular enhancing malignancy and enlarged pelvic side wall bilaterally. She also had CT scan of thorax and bone scan which were normal. She underwent robotic assisted radical anterior exenteration with Indiana pouch creation (radical cystectomy, hysterectomy, urethrectomy, and neobladder construction). The pathology of specimens revealed a 3 cm × 1.6 cm × 1.2 cm grade 2 (moderately differentiated) clear cell urethral adenocarcinoma within urethral diverticulum invading the anterior vagina with negative margins, distal bilateral ureters with negative margins, distal bilateral ureters with negative margins, 21 negative lymph nodes, and a negative hysterectomy specimen. The pathological staging was pT3N0M0. She did not have adjuvant therapy. She was well at her one-year follow-up without any recurrent disease.

Sheahan and Vega Vega [5] reported a 54-year-old woman who presented with haematuria and urethral mass on ultrasound scan. She later on had MRI scan which showed a heterogeneous mass in urethral diverticulum. She had biopsies of the mass and histological examination of the specimens revealed clear cell adenocarcinoma with clearing of the cytoplasm, moderate nuclear pleomorphism. Immunohistochemical staining revealed that the tumour cells were positive for cytokeratin 7 and p16, p53, CA125, CAM5.2, and AE1/AE3. Immunohistochemical staining also showed that the tumour cells were negative for thrombomodulin, oestrogen, progesterone, cytokeratin 20, p63, CD10, CEAP, WTI, AFP, and s100. She underwent anterior exenteration with pelvic lymphadenectomy and ileal conduit construction. Histological examination of the specimen revealed surgically clear margins but 3 out of 6 positive lymph nodes. She underwent 50.4 Gy consolidate radiotherapy due to lack of known benefit of chemotherapy in CCAU. She subsequently had PET scans which showed progression of lymphatic disease but she was alive, one year after her diagnosis.

Nakatsuka et al. [9] reported a 42-year-old woman who presented with bloody discharge from her urethra and lower back pain. Cytological examination of her urine sediment was reported to be highly indicative of adenocarcinoma. Papanicolau-stained specimens of her urine showed a small number of papillary or spherical clusters of atypical cells with many benign urothelial cells and squamous cells in the background. A few neutrophils and lymphocytes were observed; however, no necrotic debris was seen. The nuclei of the atypical cells showed an increase in the chromatin content with fine granular pattern and irregular contours; the nucleoli were prominent. Most of the atypical cells had a moderate amount of cytoplasm that was lightly stained green; however, some atypical cells showed clear, abundant cytoplasm that formed spherical clusters resembling “mirror balls.” The cytological findings were reported to be suggestive of a malignant tumour of the urinary tract system and favoured adenocarcinoma. She had computed tomography (CT) and magnetic resonance imaging scans which showed a tumour in the entire urethra. The rest of the intra-abdominal and pelvic organs and lymph nodes were normal. She had cystourethroscopy which showed two diverticula in the urethral wall in which whitish papillary and villous lesions were found. The tumours were biopsied and resected transurethrally and histological examination of the specimens was suggestive of clear cell adenocarcinoma (CCA) but was indeterminate for malignancy. She underwent total cystourethrectomy and partial resection of the vaginal wall. The final pathological diagnosis of the resected tumour was clear cell adenocarcinoma (CCA) stage III pT3N0M0.

Histological examination of the biopsy and transurethral resected specimen showed that the tumour was comprised of papillotubular lesions.

The epithelial cells which covered the tumour were cuboidal and single layered, and some of these showed a “hobnail pattern.” Most of the cells had eosinophilic cytoplasm, with the exception of a few which had clear cytoplasm. The cells that had a clear cytoplasm were positive for periodic acid-Schiff reaction. The epithelial cells exhibited relatively mild cytological atypia and they did not invade the stroma. Mitotic figures were seen at a frequency of 10/10 high-power fields. Necrotic debris was regularly seen in the lumen of the tubular structures.

Immunohistochemical staining showed that the tumour cells were positive for

- cytokeratin (CK7),
- EMA,
- carbohydrate antigen (CA) 125.

**Figure 5:**
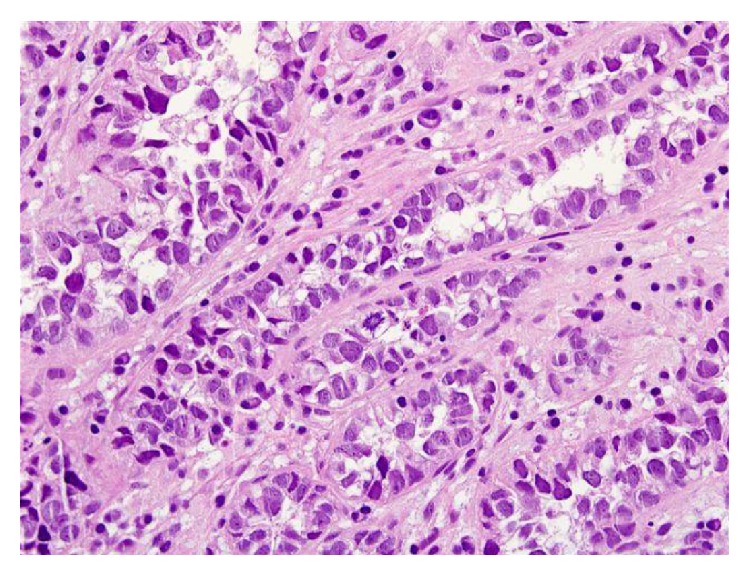
Haematoxylin and eosin staining, original magnification ×40, showing prominent cytological pleomorphism in clear cells and atypical mitotic figures. The figure was reproduced from [4, 7] with permission granted by Dr. Eddie Fridman. Copyright to Dr. Eddie Fridman. This permission is exclusive to this request specifically for this paper. Additional usage of any printed or electronic material for which Dr. Eddie Fridman holds would require copyright permission from Dr. Eddie Fridman.

Immunohistochemical staining also showed that the tumour cells were focally positive for CD15 (see Figure 5(b)).

Immunohistochemical staining showed that the tumour cells were negative for

- CK5/6,
- CK20,
- carcinoembryonic antigen,
- thrombomodulin,
- uroplakin,
- prostate-specific antigen,
- calretinin,
- oestrogen receptor,
- progesterone receptor.

The aforementioned histological findings were reported to have suggested a diagnosis of CCAU, even though definitive diagnosis of malignancy could not be elicited in view of the absence of stromal invasion in both the biopsy and the transurethrally resected specimens. Nevertheless, in the surgically resected specimens, clear atypical cells with papillotubular structure were seen to have invaded all the layers of the urethra and vaginal muscular layer. Based upon these findings the final diagnosis was CCA. The authors further reported that the Ki-67 labeling index of the tumour cells was about 20% (see Figure 5(c)) and about 5% of the tumour cells exhibited strong p53 positivity in the nucleus.

Göĝuş et al. [10] stated that CCAU is extremely rare. Most of the data on CCAU have been obtained from case reports and case series [1, 3, 10]. CCAU mostly affects females and up to half of the cases develop in urethral diverticulum [3, 11, 12].

Göĝuş et al. [10] reported a 44-year-old man with a history of two previous urinary retentions who presented with obstructive urinary symptoms. He had a rectal examination which revealed an indurated prostate. His serum prostatic-specific antigen (PSA) was 0.15 ng/mL (normal). He had cystourethroscopy which revealed a friable solid tumour in the entire urethra and bladder neck. The tumour was resected transurethrally and histological examination revealed a tumour which was composed of tubular structures lined with cells comprising hyperchromatic nuclei and clear cytoplasm. Papillary pattern was observed adjacent to these areas. Tubular and papillary structures were lined with hobnail cells in some areas of the tumour. Immunohistochemical staining of the tumour was negative for PSA. He had a CT scan of abdomen and pelvis which showed multiple bilateral internal iliac lymph nodes. He had bone scan and chest X-ray which was normal. He underwent radical cystoprostatectomy, bilateral pelvic and inguinal lymph node dissection, urethrectomy, and construction of ileal conduit. Histological examination of the cystoprostatectomy specimen confirmed features of clear cell adenocarcinoma of the bladder neck and the tumour in the entire urethra which was similar to the findings of the transurethrally resected specimens. The perivesical fat, the prostate gland, and seminal vesicles were infiltrated by the tumour. He received three cycles of methotrexate-vinblastine-epirubicin-cisplatin (MVEC) chemotherapy but he died of progressive disease 10 months after his cystoprostatectomy.

Konnak [13] in 1973 reported the first case of CCAU; since then sporadic cases of CCAU have been reported. Konnak [13] used the terminology “mesonephric carcinoma” for CCAU and postulated that the tumour perhaps emanates from the mesonephric duct or intermediate mesodermal vestiges. On the contrary, Kawano and associates [14] are of the opinion that CCAU is of origin.

Some authors [15, 16] suggested that there is a clear association between clear cell adenocarcinoma and diverticula of the urethra and that CCAU is the most common malignancy arising from diverticula of the urethra. It has been stated whilst only 10% of carcinoma of the urethra is clear cell adenocarcinoma, one-third of such carcinomas originate in a diverticulum [15].

Pollen and Dreilinger [17] in 1984 iterated their support for the homogeneity between the female paraurethral duct and the prostate gland in men based upon the finding of positive immunohistochemical staining for prostate-specific antigen (PSA) and prostatic specific acid phosphatase (PAP).

Trabelsi et al. [2] reported a 56-year-old woman who presented with visible haematuria. On examination bleeding was observed from her urethral meatus. She underwent cystoscopy which revealed a tumour protruding from the posterior urethral wall at the neck of the urinary bladder. She underwent transurethral biopsy of the tumour and histological examination of the specimen showed an invasive poorly differentiated carcinoma of the urethra. She underwent total urethrocystectomy including her anterior vaginal wall and pelvic lymph node dissection and ileal conduit construction. The bladder mucosa was normal but the tumour involved all the urethral layers. Microscopic examination of the specimen showed a tumour which was composed of nests and papillary structures which were lined with cells that had clear cytoplasm with hobnail cells in some areas of the tumour; the cells exhibited cytological atypia and high mitotic rate; the tumour cells invaded all the urethral layers but did not involve the urinary bladder. The lymph nodes were not involved. Immunohistochemical staining for prostate-specific antigen (PSA) was negative. She did not receive any adjuvant therapy and she was free of disease 3 months after her operation.

Trabelsi et al. [2] stated the following.

- Pollen and Dreilinger [17] were of the opinion that CAUs arose from the female paraurethral duct and, in their case, the tumour cells were negative for PSA.
- Zaviačič and associates [18] had reported a neoplasm with similar histological appearance and immunohistochemical features as adenocarcinoma of Skene's paraurethral gland and ducts.
- Their aforementioned findings would support the postulate of Abascal Junquera and associates [11] that the female clear cell adenocarcinoma arises from the paraurethral duct. Nevertheless, it would seem that female urethral adenocarcinoma has more than one tissue of origin with minority arising from the Skene's glands as suggested by Dodson and associates [19]. Morphologically, CCAU of the urethra must be differentiated from nephrogenic adenoma of the urethra especially on biopsy. The predominance of clear cells, severe cytological atypia, high mitotic rate, and necrosis favored the diagnosis of CCAU.
- Some authors [10, 20] stated that, in view of the rarity CCAU, the optimal treatment is not known. It would appear to be based upon the localization of the primary tumour and the presence of metastasis. Ebisuno and associates [20] stated that radical cystourethrectomy with or without irradiation was performed in most cases. Some authors [20, 21] also stated that the response to chemotherapy is also not clear.

Han et al. [22] reported a 54-year-old woman who presented with painless visible haematuria. She had vaginal ultrasound scan which revealed a sausage-like elongated mass in the urethra. Cytological examination of her voided urine revealed small clusters of rounded or papillary cells. The necrotic debris and inflammatory cells were present within some clusters of tumour cells. The tumour cells were enlarged and had abundant clear or granular cytoplasm with cytoplasmic vacuoles. The nucleus was granular and contained vesicular chromatin with prominent nucleoli. Hobnail cells and hyaline globules were also as in a histological section. They stated the following.

- The histological findings were compatible with clear cell adenocarcinoma.
- Nevertheless, cytologically, it would be necessary to make a differential diagnosis from the other adenocarcinoma or high-grade urothelial carcinoma.
- Oliva and Young [3] indicated that CCAU accounts for about 1% of male urethral carcinomas and about 15% of female urethral carcinomas.

Fridman [7] in 2011 reported an 82-year-old woman who underwent transurethral resection of a space occupying lesion which was diagnosed in her urethra and bladder neck (see Figures 1 to 11 for various microscopic and immunohistochemical characteristics of the tumour). Fridman stated that CCAUs are high-grade tumours which are common in women and macroscopically, they are papillary. Fridman summarized the features of CCAU as follows.

- CCAUs have various architectural patterns including tubules, cysts, papillae, and diffuse tumour.
- Most tumours have prominent clear cytoplasm due to glycogen and hobnailing.
- The tumour cells have prominent pleomorphism and marked cytotic activity.
- There is often muscular invasion and necrosis [23].
- The tumour cells are immunoreactive for CK7, CK903, Ki-67, and p53 and usually negative for CK20. In contrast to majority of urothelial carcinomas, they are also immunoreactive for P504S and negative for p63 [8].

Fridman [7] stated that the differential diagnosis of CCAU includes nephrogenic adenoma, a metaplastic process which often occurs in young adults with a history of genitourinary trauma, surgery, or stones. Fridman [7] further stated that, in nephrogenic adenoma, reactive changes may exist in the tumour cells as well as mitotic activity, but in such cases there is no marked pleomorphism or invasion. There are usually no clear cells in nephrogenic adenoma, and if present they are focal, and Ki-67 and p53 are usually negative or have minimal staining [24].

Fridman [7] additionally stated the following.

- Herawi et al. [25] in 2010 described a clear cell adenocarcinoma which mimicked nephrogenic adenoma due to less prominent nuclear pleomorphism, less prominent nucleoli, and fewer clear cells. Nevertheless, this variant of adenocarcinoma did exhibit extensive muscular invasion and focal hyperchromatic and pleomorphic tumour cells that would not be in nephrogenic adenoma.
- Miller and Karnes [26] in 2008 stated that clear cell adenocarcinoma is an aggressive tumour with low rates of 5-year survival. In their case, the patient had received an incomplete course of chemotherapy after she had undergone surgery. She was well during the subsequent three and half years until 5 months prior to the presentation of the case, when she underwent excision of pelvic lymph node histology of which confirmed clear cell carcinoma similar to the primary tumour. At the time of presentation of the case the patient was alive without any further recurrence.

Young and Scully [23] described the clinical and pathological characteristics of three previously unreported and 16 previously reported examples of clear cell adenocarcinoma of the urinary bladder and urethra. They stated that six of the tumours arose in the urinary bladder and 13 in the urethra. Sixteen of the patients were female, and the ages ranged between 35 years and 78 years. Most of the tumours were papillary tumours but some of the tumours were sessile. Young and Scully [23] reported that microscopic examination of the tumours showed various patterns which included tubular glands, cysts, papillae, and diffuse areas. They had identified cells with abundant glycogen-rich clear cytoplasm and hobnail cells in majority of the tumours. Young and Scully [23] advised that these tumours should be differentiated from nephrogenic adenomas. They stated the following.

- A young age or a history of genitourinary trauma, operation, or calculus may constitute a clue to the latter diagnosis; microscopic characteristics such as sheets of clear cells, significant pleomorphism, or mitotic activity would be in favour of the diagnosis of clear cell adenocarcinoma.
- The follow-up of majority of the patients, most of who underwent a radical operation, was short, but five tumours were known to have metastasized.

**Figure 12:**
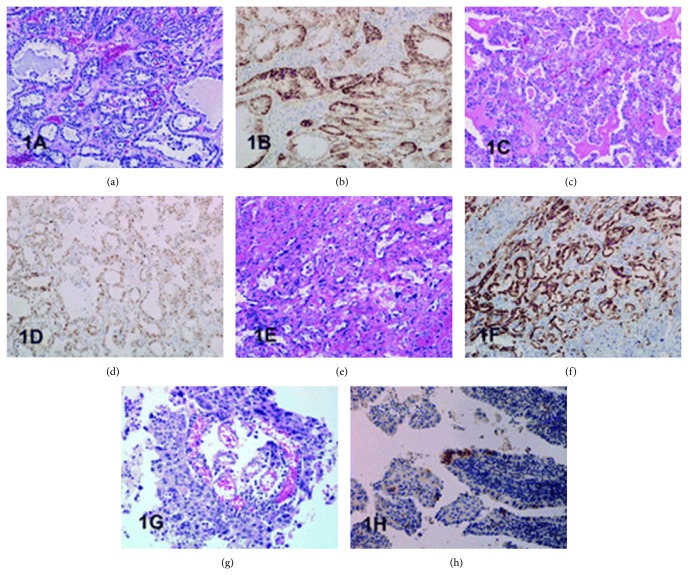
This figure shows architectural patterns of clear cell adenocarcinomas and granular cytoplasmic reactivity with P504S. (a) Tubulocystic pattern with tubules lined by cells with clear cytoplasm component (bladder case 1). (b) Positive staining with P504S bladder case 1). (c) Diffuse growth of clear cells (bladder, case 2). (d) Positive staining with P504S (bladder, case 2). (e) Tubulocystic pattern with tubules lined by hobnail cells showing moderate to severe nuclear atypia (urethra, case 3). (f) Positive staining with P504S (urethra, case 3). (g) Papillary growth pattern with papillae lined by hobnail cells with severe nuclear atypia and mitoses (urethral diverticulum, case 4). (h) Positive staining with P504S (urethral diverticulum, case 4). Haematoxylin-eosin and immunoperoxidase original magnification ×200. Reprinted from [8] Sun et al. Clear cell adenocarcinoma of the urinary bladder and urethra: another urinary tract lesion immunoreactive for P504S. Arch Pathol Lab Med 2008 Sep; 132(9): 1417–1422 reprinted with permission from Archives of Pathology and Laboratory Medicine Copyright 2008 College of American Pathologists. This permission is exclusive to this request specifically for this paper. Additional usage of any printed or electronic material for which the Archives of Pathology and Laboratory Medicine owns copyright would require permission from the editorial office.

**Figure 13:**
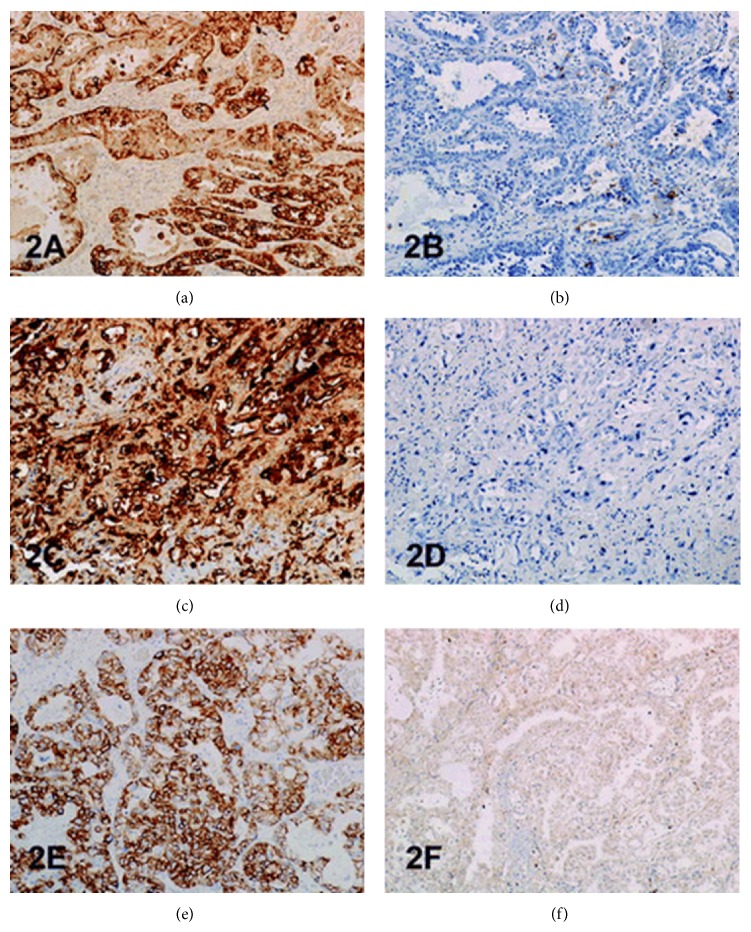
This figure shows immunoreactivity of cytokeratin (CK) 7, CK 20, K903, and p63 in clear cell adenocarcinoma. (a) Positive staining with CK7 (bladder, case 1). (b) Focally positive staining with CK20 (bladder, case 1). (c) Positive staining with CK 7 (urethra, case 3). (d) Negative staining with CK 20 (urethra, case 3). (e) Positive staining with K 903 (bladder, case 2). (f) Negative staining with p63 (bladder, case 2). Immunoperoxidase, original magnification ×200 ((a) to (e)) and ×100 (f). Reprinted from [8] Sun et al. Clear cell adenocarcinoma of the urinary bladder and urethra: another urinary tract lesion immunoreactive for P504S. Arch Pathol Lab Med 2008 Sep; 132(9): 1417–1422 reprinted with permission from Archives of Pathology and Laboratory Medicine Copyright 2008 College of American Pathologists. This permission is exclusive to this request specifically for this paper. Additional usage of any printed or electronic material for which the Archives of Pathology and Laboratory Medicine owns copyright would require permission from the editorial office.

Sun et al. [8] stated that adequate characterization had been hampered by its rarity; alpha-methyl-acyl-CoA-racemase (AMACR)/P504S had been reported to be positive in prostatic adenocarcinoma, papillary renal cell carcinoma, and gastrointestinal neoplasms; nevertheless, it had never been previously studied in clear cell carcinomas of lower urinary tract. They investigated the immunohistochemical staining profile in 4 primary clear cell carcinomas of the urinary tract including P504S. They retrieved four cases of clear cell adenocarcinoma from their archives: 2 cases from the urinary bladder (one each from a man and a woman) and 2 cases from the urethra (both from women, 1 in a diverticulum). They performed immunohistochemistry for P504S, K903, cytokeratin (CK) 7, CK20, CA 125, and p63. Sun et al. [8] reported that clear cell carcinomas had distinct immunoreactive profile: strongly positive for P504S, K903, and CK 7 and negative reactivity for p63. Two of the cases were also positive for CA 125 and CK 20 (see Figures 12 and 13 as well as Table 1 for various microscopic features and immunohistochemical characteristics of some of the tumours). Sun et al. [8] concluded the following.

- The immunohistochemical profile of clear cell carcinoma shares some similarity to conventional urothelial carcinoma; nevertheless, it deviates from urothelial carcinomas in being positive for P504S and negative for p63.
- This staining profile may indicate a nonurothelial origin for these tumours and may serve as a useful tool in the differential diagnosis of clear cell adenocarcinoma and may reflect its aetiology.
- In view of the fact that similar expression of P504S is also seen in nephrogenic adenoma, this marker should not be used to differentiate nephrogenic adenomas from clear cell adenocarcinomas.

## 4. Conclusions

Few cases of CCAU have been reported and the tumours have been reported to be aggressive with low 5-year survival rates.

CCAUs have been treated by anterior exenteration in women and cystoprostatectomy in men and at times by radiotherapy; chemotherapy has rarely been given.

## Figures and Tables

**Figure 1:**
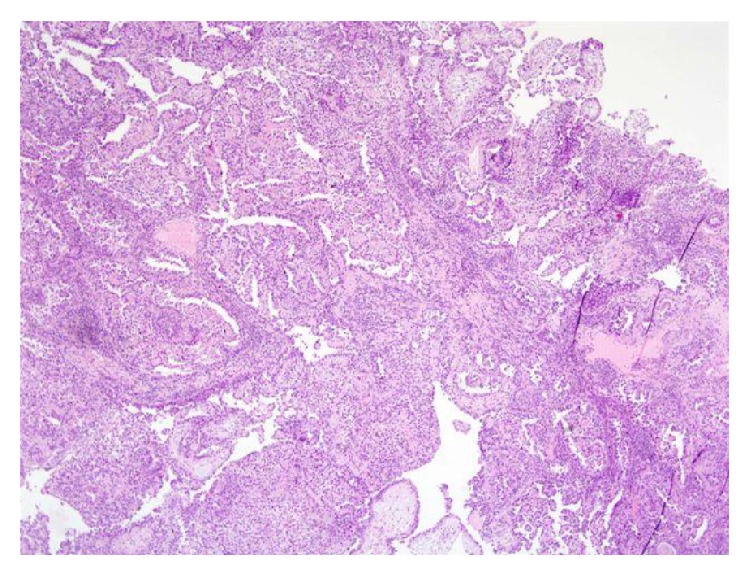
Haematoxylin and eosin staining, original magnification ×4, showing complex papillary architecture with abundant fibrovascular stroma; minimal tubular structures and focal solid areas are also seen. The figure was reproduced from [4, 7] with permission granted by Dr. Eddie Fridman. Copyright to Dr. Eddie Fridman. This permission is exclusive to this request specifically for this paper. Additional usage of any printed or electronic material for which Dr. Eddie Fridman holds would require copyright permission from Dr. Eddie Fridman.

**Figure 2:**
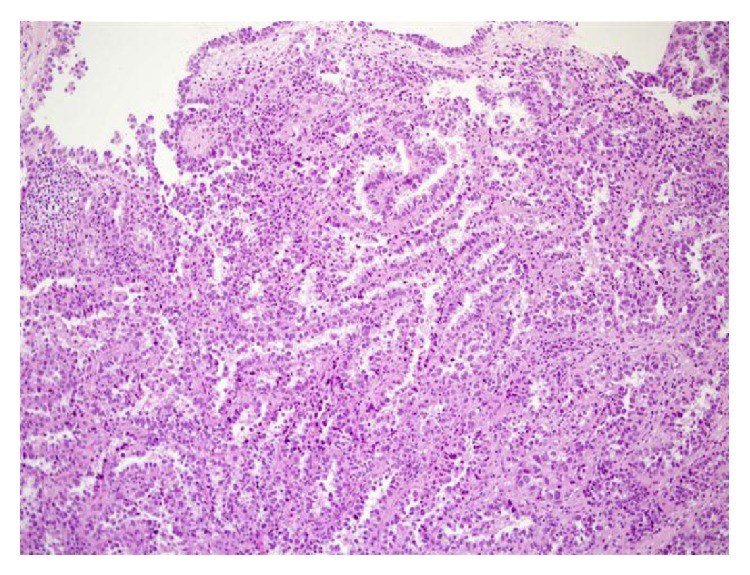
Haematoxylin and eosin staining, original magnification ×10, showing papillary and tubular structures, with cytologically atypical epithelial lining with clear cells and focal hob-nail appearance. The figure was reproduced from [4, 7] with permission granted by Dr. Eddie Fridman. Copyright to Dr. Eddie Fridman. This permission is exclusive to this request specifically for this paper. Additional usage of any printed or electronic material for which Dr. Eddie Fridman holds would require copyright permission from Dr. Eddie Fridman.

**Figure 3:**
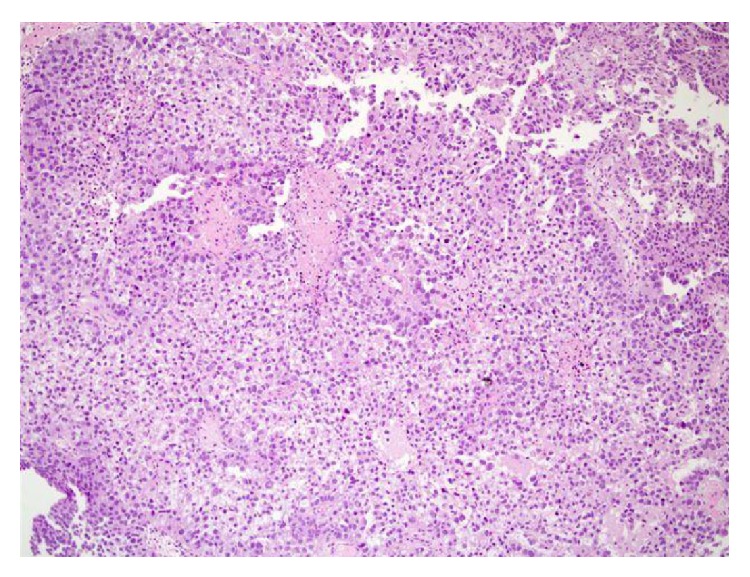
Haematoxylin and eosin staining, original magnification ×10, showing areas of solid growth pattern composed of atypical clear cells. The figure was reproduced from [4, 7] with permission granted by Dr. Eddie Fridman. Copyright to Dr. Eddie Fridman. This permission is exclusive to this request specifically for this paper. Additional usage of any printed or electronic material for which Dr. Eddie Fridman holds would require copyright permission from Dr. Eddie Fridman.

**Figure 4:**
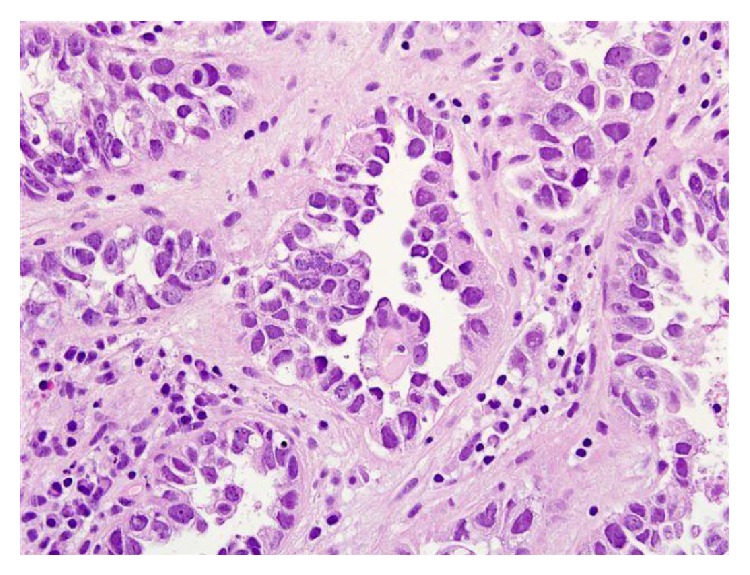
Haematoxylin and eosin staining, original magnification ×40, high magnification, showing prominent cytological nuclear atypia and few eosinophilic globules. The figure was reproduced from [4, 7] with permission granted by Dr. Eddie Fridman. Copyright to Dr. Eddie Fridman. This permission is exclusive to this request specifically for this paper. Additional usage of any printed or electronic material for which Dr. Eddie Fridman holds would require copyright permission from Dr. Eddie Fridman.

**Figure 6:**
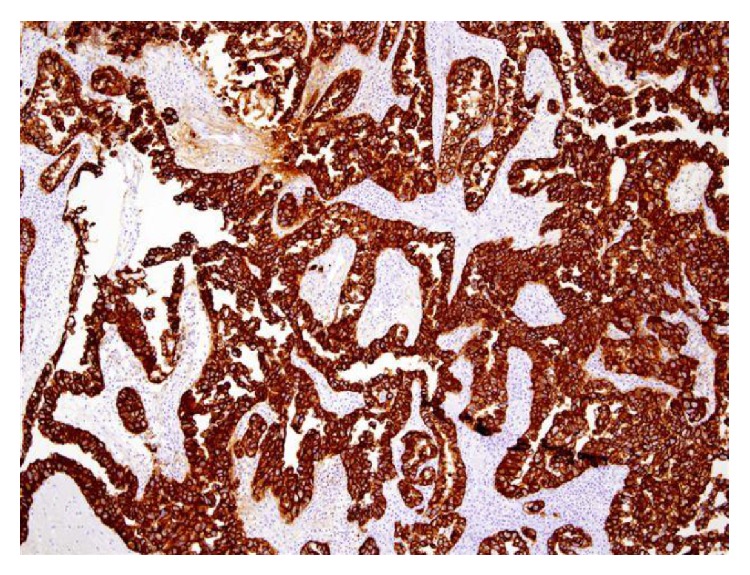
Immunohistochemical staining of clear cell adenocarcinoma showing strongly positive staining for CK7. The figure was reproduced from [4, 7] with permission granted by Dr. Eddie Fridman. Copyright to Dr. Eddie Fridman. This permission is exclusive to this request specifically for this paper. Additional usage of any printed or electronic material for which Dr. Eddie Fridman holds would require copyright permission from Dr. Eddie Fridman.

**Figure 7:**
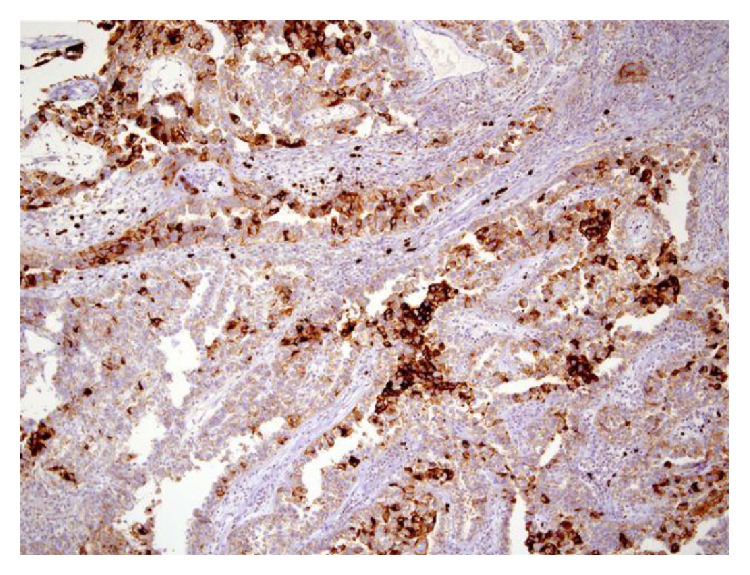
Immunohistochemical staining for clear cell adenocarcinoma showing positive staining for CD15. The figure was reproduced from [4, 7] with permission granted by Dr. Eddie Fridman. Copyright to Dr. Eddie Fridman. This permission is exclusive to this request specifically for this paper. Additional usage of any printed or electronic material for which Dr. Eddie Fridman holds would require copyright permission from Dr. Eddie Fridman.

**Figure 8:**
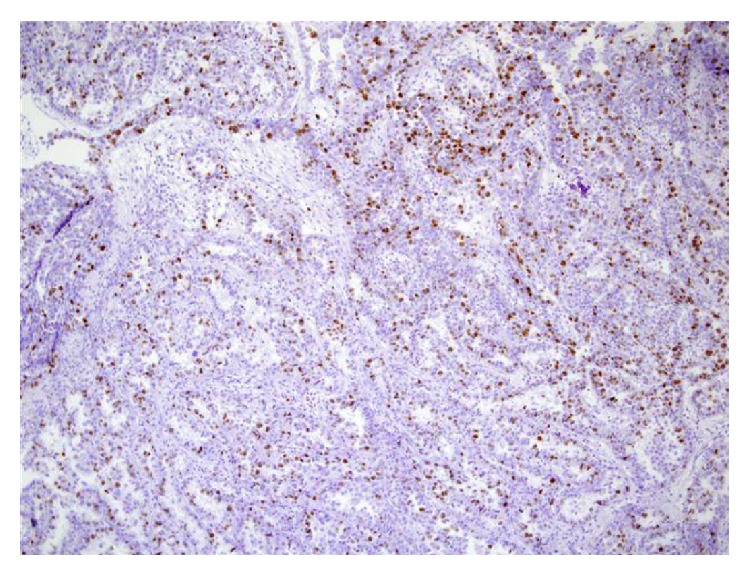
Immunohistochemical staining of clear cell adenocarcinoma showing positive staining for Ki-67 (original magnification ×10). The figure was reproduced from [4, 7] with permission granted by Dr. Eddie Fridman. Copyright to Dr. Eddie Fridman. This permission is exclusive to this request specifically for this paper. Additional usage of any printed or electronic material for which Dr. Eddie Fridman holds would require copyright permission from Dr. Eddie Fridman.

**Figure 9:**
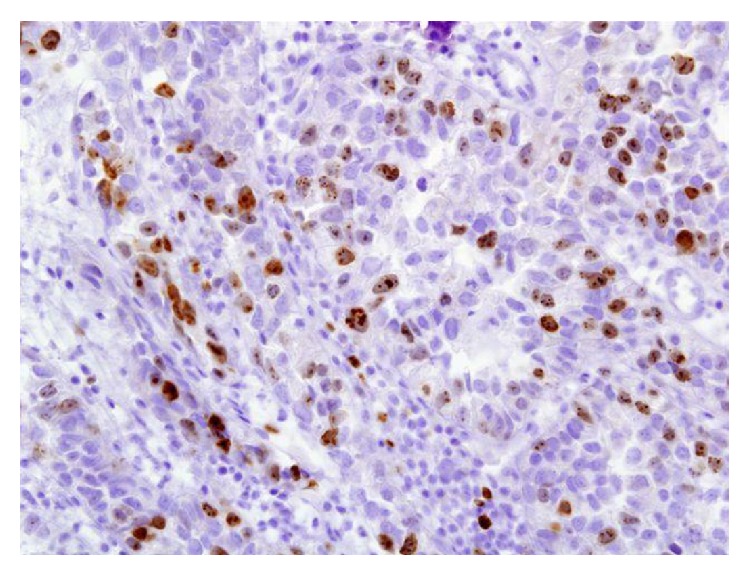
Immunohistochemical staining of clear cell adenocarcinoma showing positive staining for Ki-67 (original magnification ×40). The figure was reproduced from [4, 7] with permission granted by Dr. Eddie Fridman. Copyright to Dr. Eddie Fridman. This permission is exclusive to this request specifically for this paper. Additional usage of any printed or electronic material for which Dr. Eddie Fridman holds would require copyright permission from Dr. Eddie Fridman.

**Figure 10:**
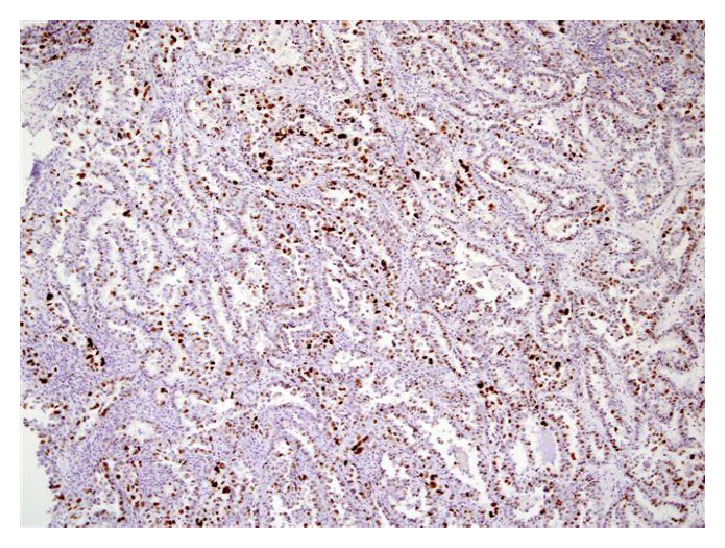
Immunohistochemical staining of clear cell adenocarcinoma showing positive staining for p53 (original magnification ×10). The figure was reproduced from [4, 7] with permission granted by Dr. Eddie Fridman. Copyright to Dr. Eddie Fridman. This permission is exclusive to this request specifically for this paper. Additional usage of any printed or electronic material for which Dr. Eddie Fridman holds would require copyright permission from Dr. Eddie Fridman.

**Figure 11:**
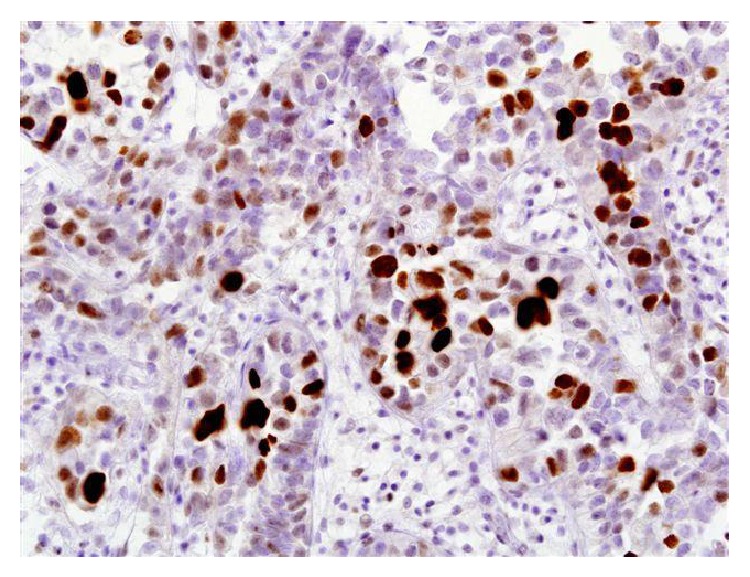
Immunohistochemical staining of clear cell adenocarcinoma showing positive staining for p53 (original magnification ×40). The figure was reproduced from [4, 7] with permission granted by Dr. Eddie Fridman. Copyright to Dr. Eddie Fridman. This permission is exclusive to this request specifically for this paper. Additional usage of any printed or electronic material for which Dr. Eddie Fridman holds would require copyright permission from Dr. Eddie Fridman.

**Table 1:** This table shows immunohistochemical staining characteristics of clear cell carcinoma. Reproduced from Sun et al. [8]. Clear cell adenocarcinoma of the urinary bladder and urethra: another urinary tract lesion immunoreactive for P504S. Arch Pathol Lab Med 2008 Sep; 132(9): 1417–1422 reprinted with permission from Archives of Pathology and Laboratory Medicine Copyright 2008 College of American Pathologists. This permission is exclusive to this request specifically for this paper. Additional usage of any printed or electronic material for which the Archives of Pathology and Laboratory Medicine owns copyright would require permission from the editorial office.

Case number	P504S	CK7	CK20	CA 125	K903	p63
1	+++	+++	++	++	+++	0
2	+++	+++	++	+	+++	0
3	+++	+++	0	0	+++	0
4	+	+++	0	++	+++	0

^*^CK indicates cytokeratin; 0, less than 5% of tumor cells staining positive; +, 5% to 25%; ++, 26% to 50%; and +++, greater than 50%.
